# Health Screening Strategies for Artisanal and Small-Scale Miners for Tuberculosis, Human Immunodeficiency Virus and Silicosis: A Case of the USAID-Supported Kunda Nqob’iTB Project in Zimbabwe

**DOI:** 10.3390/ijerph21010070

**Published:** 2024-01-08

**Authors:** Dingani Moyo, Fungai Kavenga, Florence Moyo, Orippa Muzvidziwa, Godknows Madziva, Blessings Chigaraza, Mpokiseng Ncube, Precious Madadangoma, Hellen Masvingo, Tafadzwa Charity Muperi, Tariro Christwish Mando, Ronald Thulani Ncube

**Affiliations:** 1 Baines Occupational Health Services, Harare 024, Zimbabwe; florencem@bainesohs.org (F.M.); orippam@bainesohs.org (O.M.); godknowsm@bainesohs.org (G.M.); blessingsc@bainesohs.org (B.C.); mpokisengn@bainesohs.org (M.N.); preciousm@bainesohs.org (P.M.); hellenm@bainesohs.org (H.M.); tafadzwam@bainesohs.org (T.C.M.); 2 Department of Community Medicine, Faculty of Medicine, National University of Science and Technology, Bulawayo 029, Zimbabwe; 3 School of Public Health, University of the Witwatersrand, Johannesburg 2193, South Africa; 4 Ministry of Health and Child Care, Harare 024, Zimbabwe; drkav8@gmail.com (F.K.); tariro.christwish@gmail.com (T.C.M.); 5 Department of Health Sciences, Faculty of Health, Zimbabwe Open University, Gweru 054, Zimbabwe; 6 The Union Zimbabwe Trust, Harare 024, Zimbabwe; rncube@uzt.org.zw

**Keywords:** artisanal miners, tuberculosis, silicosis, occupational health, human immunodeficiency virus

## Abstract

Artisanal and small-scale mining is characterized by excessive exposure to physical, chemical, ergonomic, psychosocial and biological hazards. There is a high burden of tuberculosis (TB), human immunodeficiency virus (HIV) infections and silicosis among artisanal and small-scale miners (ASMs). The aim of this project report is to describe lessons learned from strategies implemented to reach ASMs with screening services for TB, HIV and silicosis in Zimbabwe through the Kunda-Nqob’i TB (KNTB) project supported by the United States Agency for International Development (USAID). The intervention package for screening ASMs for TB, HIV and silicosis included service provision through two occupational health clinics at two provincial hospitals and a mobile workplace-based screening (WBS) facility at the mining sites. From 1 October 2020 to 30 September 2023, 10,668 ASMs were screened, with a high number of cases of silicosis (21%) and TB (7.4%). There was a high burden of HIV (30%) in ASMs attending the occupational health clinics. The two occupational health clinics screened 3453 ASMs, while the mobile WBS activities screened 7215 ASMs during the period. A total of 370 healthcare workers (doctors/clinical officers, nurses, environmental health technicians and district tuberculosis and Leprosy control officers) were trained on TB and the fundamental diagnostic principles of silicosis. The KNTB project has been successful in reaching out to many ASMs operating in remote and hard-to-reach mining areas. The KNTB project has brought to light the positive health-seeking behavior of ASMs operating in remote areas. The project has brought to the fore the effectiveness of multi-stakeholder engagement and collaboration in reaching out to ASMs in remote areas with health screening services. There is a high burden of TB, HIV and silicosis in ASMs. Screening for TB, HIV and silicosis using workplace-based screening and occupational health clinics is an effective strategy and should be rolled out to all areas with high artisanal and small-scale mining activity.

## 1. Background

Zimbabwe has over 500,000 artisanal and small-scale miners (ASMs) and about 2 million people who depend on them [1]. According to the latest research, there is a high burden of tuberculosis (TB) (7%), silicosis (19%) and human immunodeficiency virus (HIV) (18%) among ASMs in Zimbabwe [2]. ASMs are highly mobile and operate in hard-to-reach areas using archaic mining methods, with near-absent access to health services [3,4]. Zimbabwe has not yet implemented the basic occupational health services model that integrates occupational health services into primary healthcare. Apart from two Kunda Nqobi’TB (KNTB) project occupational health clinics, there are no other occupational health clinics in most public health institutions in Zimbabwe. There are very limited human resources with expertise or training in occupational health. Artisanal and small-scale mining is associated with several occupational health conditions, which include, but are not limited to, the following: mercury intoxication, musculoskeletal disorders, noise-induced hearing loss, malaria and silicosis [5,6,7,8,9,10,11]. Silicosis is among the most common occupational respiratory conditions among ASMs [2]. Silicosis confers a three- to four-fold risk of TB infection, while HIV increases the risk of TB infection by almost five- to six-fold [5,12,13,14,15]. Furthermore, those with silicosis and HIV have a multiplicative risk of TB infection in excess of fifteen-fold [5,15]. In addition, miners exposed to silica dust with sub-radiological silicosis, even in the absence of silicosis, are at an increased risk of TB infection.

Silicosis is a serious occupational lung disease that is permanent and irreversible [12,14,16]. Exposed ASMs with sub-radiological silicosis require early identification and removal from further silica-dust exposure. Biomarkers such as clara cell protein 16 (CC-16) have been reported to be useful in the early diagnosis of sub-radiological silicosis and should be considered in exposed ASMs [17,18]. Silicosis is a compensable disease in Zimbabwe among workers who are formally employed in organizations that contribute to the National Social Security Authority’s Compensation Schemes. Most ASMs are informal and are not covered by the National Compensation Scheme. In Zimbabwe, there is no routine system for lung autopsy for workers or miners exposed to silica dust. ASMs lack basic occupational health services and occupational hygiene services. There are no occupational hygiene assessments for silica dust that are performed in artisanal and small-scale mining.

The Ministry of Health and Child Care (MoHCC) in Zimbabwe, in collaboration with Baines Occupational Health Services (BOHS) through the United States Agency for International Development (USAID)-funded KNTB project, has been screening ASMs for TB, HIV and silicosis since 2020. The KNTB project is being implemented in the following districts in Zimbabwe: Gweru, Shurugwi, Zvishavane, Kwe Kwe, Chirumhanzu, Insiza, Mwenezi and Gwanda.

The determinants of health in artisanal and small-scale miners and of the ASMs’ health needs in Zimbabwe include, but are not limited to, the following: living conditions, nutrition and hygiene; safe work environment and mining processes; financial support; healthcare services; and formalization and education [19]. ASMs face several challenges regarding diagnosis, treatment and follow-up in Zimbabwe. In Midlands Province, the five project districts have 185 health facilities (Chirumhanzu 22, Gweru 47, Kwe Kwe 51, Shurugwi 35 and Zvishavane 30) [20]. Furthermore, in Masvingo Province, Mwenezi District has 24 health facilities, while in Matabeleland South Province, Insiza District has 20 and Gwanda District has 32 health facilities. With the exception of Gwanda District and Gweru District, none of the health facilities in these districts have any specific or dedicated occupational health clinics for ASMs. ASMs in the KNTB project districts have cited long distances to health facilities, lack of financial resources for medical fees and the occasional shortage of medicines at health facilities as key barriers to accessing occupational health services [21]. Regarding TB treatment in some of the KNTB project districts, Matabeleland South Province had the lowest treatment success rate of 77.3% and a high death rate of 30.0%, while Masvingo had a death rate of 27.3% [22]. Furthermore, pre-diagnosis loss to follow-up, TB treatment loss to follow-up and high death rates are prevalent in provinces with artisanal and small-scale mining activities in Zimbabwe. The main aim of this project report is to describe lessons learned from providing TB, HIV and silicosis screening services to ASMs in Zimbabwe. The specific objectives of this project report are to (i) describe the strategies employed in providing health services to ASMs, (ii) describe the approach of demand creation for services and (iii) provide motivation for improved access and screening for TB, HIV and silicosis in all districts with high artisanal and small-scale mining activity in Zimbabwe.

## 2. Strategic Approach to Delivering Health Services to Artisanal and Small-Scale Miners

### 2.1. KNTB Project Description

The KNTB program is a 5-year Cooperative Agreement (1 October 2019 to 30 September 2024) between the United States Agency for International Development (USAID) and the Union Zimbabwe Trust (UZT) under the TB Local Organization Network (TB-LON) funding mechanism [23]. TB-LON is part of USAID’s Global Accelerator Initiative to end TB globally. Through the TB-LON, USAID funds local organizations in priority countries to implement locally generated solutions to improve TB prevention, diagnosis and care services. The Union Zimbabwe Trust is the lead partner in Zimbabwe and implements the program through a consortium of three other local organizations, namely, BOHS, Hospice and Palliative Care Association of Zimbabwe (HOSPAZ) and Jointed Hands Welfare Organization (JHWO). The overall project aim is to find missing TB cases, particularly among high-risk groups, link them to care and treat them successfully. ASMs exposed to silica-containing dust, children, people with malnutrition, diabetes and/or HIV, among several other risk factors, are more susceptible to TB infection [13,14,24,25].

The UZT is responsible for implementing specific TB health-system-strengthening-related interventions. JHWO supports community-related interventions in TB case finding, prevention, patient care support and demand creation for TB services. HOSPAZ supports the integration of a palliative care package within the primary healthcare delivery system with TB management for improved quality of care, while BOHS supports the institutionalization of occupational health services for routine TB screening. BOHS also screens ASMs for HIV and silicosis, which are important and highly prevalent risk factors for TB infection among ASMs. This is implemented through mobile workplace-based screening (WBS) services in remote mining sites and two dedicated occupational health clinics within provincial public health institutions in Gwanda and Gweru. These two strategies for screening ASMs were selected based on the high mobility of ASMs in mining operations that are in remote and hard-to-reach areas and underserved by healthcare facilities. Mobile WBS services were designed to access remote and hard-to-reach areas and provide screening services at several mining sites. The two dedicated occupational health clinics were designed to offer free services to ASMs and reduce patient waiting times. There were no dedicated or specialized occupational health clinics in the KNTB project districts for ASMs prior to project implementation.

### 2.2. BOHS-Supported Activities for ASMs

BOHS’ role in the KNTB project is to screen for TB, HIV and silicosis among ASMs in the eight districts. In collaboration with the MoHCC, BOHS implemented three strategies aimed at improving access to quality TB, HIV and silicosis screening services for ASMs. The strategies included screening through dedicated occupational health clinics and WBS services. The third strategy included the capacity building of health workers in the supported project districts to improve the quality of their skills in demand creation activities as well as managing ASMs presenting for TB, HIV and silicosis screening services.

#### 2.2.1. Dedicated Occupational Health Clinics (OHCs)

BOHS supported the establishment of two dedicated OHCs that are integrated into two public health institutions, Gwanda and Gweru provincial hospitals in Matabeleland South and Midlands Provinces, respectively. The OHCs were set up at provincial health facilities with high volumes of ASMs in the two provinces. The two OHCs integrated into public health institutions and run by MoHCC staff are the first of their kind in Zimbabwe. This approach is an improvement of the previous OHC (not operational now) that was established as a stand-alone facility operated solely by the Wits Health Consortium staff at Kadoma General Hospital in Zimbabwe, supported by the Global Fund TB in the Mining Sector project. The OHCs are specifically dedicated to screening ASMs for TB, HIV and silicosis for free. The clinics are supported by the KNTB project exclusively for ASMs and offer free TB, HIV and silicosis screening using existing hospital infrastructure, including healthcare workers with technical support from BOHS project staff. The support from the KNTB project comes in the form of basic occupational health equipment (audiometers, spirometers, consumables) and other medical equipment and furniture for designated consultation rooms, nurses’ screening rooms and reception. The OHCs are well equipped and manned by healthcare workers from the provincial hospitals. Seconded project staff include two occupational health nurse practitioners and sessional occupational health physicians from BOHS.

The two clinics offer free TB, HIV and silicosis screening services to ASMs. Audiometry, spirometry, laboratory and radiology services are offered for free to all ASMs. All ASMs are assessed for TB and silicosis using a symptom-based screening tool, chest radiograph and clinical evaluation. Audiometry and spirometry testing are conducted based on clinical indications. All cases diagnosed with silicosis are supposed to undergo spirometry except where the test is contraindicated. The diagnosis of TB is based on a positive result in the Xpert mycobacterium tuberculosis and resistance to rifampicin (MTB/RIF), Ultra/Truenat MTB and MTB Plus assays or clinical findings as per the National TB Guidelines [26]. A diagnosis of silicosis is based on the radiological appearance of nodules in both lung fields with or without symptoms and is made after excluding active TB. A diagnosis of silico-TB is based on the presence of both silicosis and TB diagnoses. All healthcare staff working at the OHCs underwent training on the fundamentals of occupational health and diagnostic skills for silicosis and TB. ASMs are either referred by their peers, self-referred or referred by other health facilities to the OHCs for TB, HIV and silicosis screening services. ASM logistic officers, under the supervision of the project staff and miners’ associations, sensitize and mobilize their peers in their respective districts to go for screening at the OHCs and WBS services. The OHCs support and follow-up ASMs identified during WBS services in remote areas.

Data and information on ASMs are recorded as part of routine hospital services. Data are collected through an occupational health examination form, where the demographic data, clinical information and the results of investigations such as sputum results, chest-X-ray results and HIV results, among other clinically indicated tests, are recorded. Data are recorded in the health facility’s daily attendance registers, TB registers and TB preventive therapy (TPT) registers. The local health institution’s daily attendance register captures the diagnoses, laboratory test results and radiology results. ASMs diagnosed with HIV and/or silicosis who are commenced on TPT are entered in the TPT register. All diagnosed TB cases are entered in the TB register at the local occupational health clinic. All collected data and information are entered into the MoHCC health information management system. Data are then imported manually into an Excel sheet for cleaning and coding. On a weekly basis, data are imported into an Excel sheet for analysis, and the results are presented in graphs and tables using Microsoft Excel version 2019 (Microsoft Corporation, Albuquerque, NM, USA).

#### 2.2.2. Workplace-Based Screening (WBS) Services for ASMs

ASMs are highly mobile and operate in very remote areas away from set health facilities, and BOHS therefore supports WBS services at remote artisanal and small-scale mining sites. This set-up encourages the ASMs to receive services on time as compared to accessing the existing health services in their districts, which are often far away, short-staffed and congested. This is one of the first strategies of its kind in Zimbabwe among ASMs that utilizes a unique onsite workplace-based occupational health approach to TB, HIV and silicosis screening. The screening strategy improves on the current community-based screenings and introduces a workplace-based screening approach targeting ASMs exposed to occupational hazards such as silicosis and/or silica-dust exposure. Previous and other current screening initiatives in Zimbabwe have focused on community screening, including mining communities. The KNTB approach specifically focuses on, among other important issues, silicosis, silica-dust exposure and HIV as important risk factors for TB infection. BOHS utilizes an occupational health approach that is workplace-based and not community-based. WBS activities are performed quarterly across the eight project districts with high artisanal and small-scale mining activity. The MoHCC provides human resources, consumables and laboratory services for the WBS activities. The intervention consists of setting up screening onsite or close to mining sites. Site preparations include setting up multiple workstations to optimize client flow. Hired mobile ablution facilities, a generator, furniture, basic equipment and other consumables are mobilized as part of WBS at or close to the artisanal and small-scale mining sites.

Each WBS activity is coordinated by the provincial medical office and lasts for four days. The MoHCC, through the local health facility, is responsible for the follow-up and management of all screened ASMs. The services offered during WBS include screening for TB, HIV, silicosis and other medical conditions. During the screening sessions, vital observations, such as blood pressure, weight and temperature, are checked. Basic education and counseling on TB, HIV, silicosis and other medical conditions are offered during the screening process.

The data collection process is as described above under OHCs. For WBS services, registers of the local MoHCC clinic in the district nearest to the ASM screening sites are used. This allows for continued care and follow-up by the local clinic after the WBS activities. At the end of each WBS activity, data are entered into MS Excel for analysis.

#### 2.2.3. Demand Creation for WBS and OHCs

The KNTB project uses a key stakeholder engagement and participatory strategy for engaging ASMs to participate in TB, HIV and silicosis screening. The WBS services are preceded by mobilization activities to sensitize ASMs and their leadership. The main goal of demand creation is to reach as many ASMs across several mining sites in both remote and peri-urban areas to increase access to screening services. The pre-WBS visits provide an opportunity to identify no more than four screening sites (artisanal and small-scale mines) based on the anticipated reach of ASMs, accessibility, and permission from local ASM leadership and the responsible local urban or rural authorities.

The strategy employed in mobilization includes engaging ASM leadership, as well as local traditional and community leadership around the mining sites. It includes establishing rapport with ASMs and getting them to lead the mobilization exercises of their peers. ASM leadership consultation, collaboration and participation in demand creation is a critical part of the KNTB project’s strategy in engaging the ASM constituency. Consultation with district local authorities assists in gaining acceptance of the screening program within the mining areas, and it encourages the building of trust.

The KNTB project demand creation process is undertaken over two days at least a week prior to WBS. The demand creation visit is conducted by a team of BOHS staff, MoHCC TB focal persons, representatives of the ASM associations’ leadership and program officers from JHWO. The team visits the different mining sites to conduct several activities prior to WBS. These activities include, but are not limited to, the following: (i) informing the ASMs about the forthcoming WBS, describing the process, benefits and key logistics for screening, (ii) distributing posters and pamphlets regarding the event, (iii) establishing rapport with the ASMs and (iv) assessing the road infrastructure and suitable camping sites for WBS. These visits serve to identify ASMs who would assist the WBS team with control and order during the WBS. Social mobilization for planned WBS is amplified through local health facilities, ASM leadership, local traditional leadership structures and other implementing partners in the artisanal and small-scale mining sites.

Demand creation is also carried out for the OHCs. This is achieved by collaborating with ASM associations, such as Youths in Mining, Women in Mining, special syndicates/cooperatives and the ASM community in general. Key ASM logistic officers were identified by the project from among active miners to effectively mobilize their peers for screening purposes. The ASM logistic officers encourage, educate and coordinate their peers to attend screening sessions at both the OHCs and the WBS sessions. Further mobilizations for services happen through the social media platforms of ASMs and through other implementing partners working with ASMs on other projects, such as HIV projects.

#### 2.2.4. Capacity Building on TB and Occupational Lung Diseases

The KNTB project introduced capacity building for health workers to improve the quality of TB, HIV and silicosis services provided to ASMs. Capacity building focuses on diagnostic and management skills for TB, HIV and silicosis. Doctors, nurses and environmental health practitioners undergo specific training courses to improve their skills in screening ASMs. During the period under review, the project supported the capacity building of health workers at public healthcare institutions on TB, HIV and silicosis. The training was offered to nurses, doctors and environmental health practitioners. Nurses underwent training on the fundamentals of occupational health to equip them with diagnostic and management skills on TB, HIV and silicosis. The project also supports four-day training courses for doctors on the fundamentals of occupational health and diagnostic skills on TB, HIV and silicosis. The other training courses for doctors include a four-day introduction to the International Labour Organization International Classification of Radiographs of Pneumoconioses. Participants were selected by the district health executives from local health institutions from across the eight supported project districts.

## 3. Results

Onsite screening at the mining sites and at the dedicated OHCs provided a good opportunity for free screening for TB, HIV and silicosis among ASMs. There was a very high demand for TB, HIV and silicosis screening services at both the mining sites and at the OHCs. Table 1 below shows the overall data for WBS and Occupational Health Clinic activities. From 1 October 2020 to the end of September 2023, the OHCs and WBS facility screened 10,668 ASMs for TB and HIV. Seven hundred and ninety-three (7.4%) cases of TB were diagnosed, among whom 314 (40%) were bacteriologically confirmed. Of the 10,668 ASMs attended to, 10,282 ASMs were screened for silicosis, and 2150 (21%) were diagnosed with silicosis. Among those diagnosed with silicosis, 1665 ASMs were eligible for TPT, and only 1212 (73%) were successfully initiated on TPT. The high mobility of ASMs and their operation in remote and hard-to-reach areas posed challenges to TPT initiation. There were fewer ASMs screened for silicosis compared to TB due to challenges with radiology services as a result of power outages or X-ray maintenance services.

The overall rate of bacteriologically confirmed TB cases in the three years was 40%, well below the reported WHO average for 2022 of 63% [27]. There was an upward trend in the number of bacteriologically confirmed TB cases over the three years of project implementation, reaching 48% in the period from 1 October 2022 to the end of September 2023. However, the proportion of bacteriologically confirmed TB cases during this period was lower when compared to the 57% attained overall in the KNTB-supported districts [28]. This could have been partly due to the use of microscopy testing for TB following occasional stock-outs of Gene Xpert cartridges during the period. At the WBS, apart from those who tested HIV-positive, data were not recorded on the total number of ASMs who underwent HIV testing or those who knew their HIV status.

### 3.1. Occupational Health Clinics and Workplace-Based Screenings

Table 2 and Table 3 below show the numbers of ASMs screened at the OHCs and WBS services, respectively, from 1 October 2020 to the end of September 2023. The WBS services attended to twice as many ASMs as compared to the OHC screening services. The OHC screening activities diagnosed more TB and silicosis cases than the WBS services. The OHCs screened 3453 ASMs for TB, and 667 (19.3%) were diagnosed with TB, among whom 265 (40%) were bacteriologically confirmed (Table 2). Furthermore, 3305 ASMs were screened for silicosis, and 1056 (32%) ASMs were diagnosed with silicosis through the OHCs. Of the 641 ASMs with silicosis who were eligible for TB preventive therapy (TPT), only 434 (68%) were successfully initiated on TPT. In addition, 1904 (55%) ASMs had recorded HIV results, of which 578 (30%) were HIV positive.

The number of ASMs screened at the OHCs in the second year doubled when compared to the first year of screening, 2020 to 2021. However, there was a decrease in the number of ASMs screened through WBS during the period from October 2022 to September 2023. This was due to fewer WBS activities that were conducted when compared to the period from October 2021 to September 2022. Furthermore, this could have been due to more ASMs utilizing the two OHCs for screening purposes.

Table 3 shows that the TPT initiation rate was higher (76%) at WBS services compared to OHCs (68%). The proportion of bacteriologically confirmed TB was almost the same for the OHCs and WBS. The TB yields at the OHCs were almost five times higher than at WBS services despite the WBS services accessing double the number of ASMs. Silicosis yields at the OHCs were double those diagnosed at the WBS services. This is most likely due to the fact that most ASMs who are sick are more likely to visit the OHCs, whereas WBS provides access to active miners, who are more likely to be healthier than those presenting at the OHCs.

### 3.2. Capacity Building on TB, HIV and Silicosis

During the period from 1 September 2019 to 30 September 2023, 370 health workers and ASMs (183 males and 187 females) were trained on TB, HIV and silicosis through the KNTB project. These included 84 doctors, 167 nurses, 102 environmental health technicians and 17 ASMs.

## 4. Support Services

The support services under BOHS play a crucial role in project implementation. The support services include Safety, Health, Environment and Quality (SHEQ) technical services, monitoring and evaluation, and administration and finance services. SHEQ technical support ensures that BOHS implements its activities while adhering to good standards of health, safety and environmental protection. For all activities that include screening at OHCs and WBS services, SHEQ technical services ensure that hazard identification and risk assessments (HIRAs) are conducted, and for WBS, environmental impact assessments are performed to ensure that the project activities are environmentally friendly. A monitoring and evaluation technical expert provides support to BOHS services and tracks the performance of the project against the set performance indicators.

Administrative support for the project is rendered by the administrative and finance team of the project under BOHS. The coordination of transport logistics, the organization of ablution facilities, the sourcing of consumables, the coordination of key stakeholder communication networks, the organization of logistics for laboratory investigations, supporting health facilities and logistics for the follow-up of ASMs are some of the functions that are carried out by the administrative team. The support services are also involved in the coordination and movement of occupational health equipment and other supporting equipment during the WBS services.

## 5. Lessons Learned

There are several lessons learned from the implementation of the KNTB project, which include the following:

i. The burden of TB among ASMs in the project districts of 7400 per 100,000 is 39 times higher than that in the general population (190 per 100,000) in Zimbabwe [29].
ii. The silicosis burden is high at 21% among ASMs who were screened at both the OHCs and WBS services.
iii. TPT initiation among ASMs with silicosis is low at 73% and remains a challenge due to the high mobility and operation in remote and hard-to-reach areas, which makes follow-up for initiation a challenge.
iv. A very high proportion of ASMs (45%) attending the OHCs have an unknown HIV status.
v. The proportion of ASMs with TB who have a bacteriologically confirmed diagnosis is low at 40%. Distinguishing TB and silico-TB from silicosis poses a huge diagnostic challenge due to similarities in radiological appearances. The low proportion of bacteriologically confirmed TB cases requires further exploration.
vi. WBS services reach out to a higher number of ASMs in remote areas, almost double, when compared to OHCs, although the TB and silicosis yields are lower.
vii. Using a collaborative multi-stakeholder approach that includes ASM leadership to mobilize ASMs for screening services is effective in reaching out to ASMs with occupational health services.

## 6. Recommendations

The following recommendations drawn from the implementation of the KNTB project are worth noting:

i. The high demand and prevalence of TB (7.4%), silicosis (21%) and HIV (30%) among ASMs present a strong case for the replication and scaling-up of the KNTB project in other districts in Zimbabwe with high artisanal and small-scale mining activity.
ii. The KNTB project has demonstrated that TPT initiation levels (73%) among ASMs pose a challenge. A targeted TPT program for ASMs with silicosis and/or HIV should be designed to cater to all ASMs who have high mobility and operate in remote areas.
iii. The number of ASMs with a known HIV status at the OHCs is very low at 55%. It is recommended that a strong campaign on voluntary counseling and testing be rolled out among artisanal and small-scale mining areas. Furthermore, it is recommended that data on the HIV status of all ASMs be collected.
iv. There is a need to follow up on the low numbers of bacteriologically confirmed TB cases. Capacity building on TB and silicosis diagnosis and management should be increased to overcome the diagnostic difficulties in distinguishing between the two conditions.
v. In addition to chest radiographs, the MoHCC in Zimbabwe should also consider introducing testing for the biomarker CC-16 during the medical surveillance of all silica-dust-exposed ASMs.
vi. National policy strategies on healthcare delivery systems for ASMs should integrate occupational health services into primary healthcare services, especially in districts with high artisanal and small-scale mining activity.

## 7. Conclusions

The USAID-funded KNTB project has succeeded in accessing ASMs and offering TB, HIV and silicosis screening services. The WBS approach, together with the dedicated OHCs for ASMs, has been an effective strategy in identifying and managing ASMs suffering from TB, HIV and silicosis. Through this approach, ASMs have been able to receive early diagnoses and treatments, resulting in improved health outcomes. There is a need to replicate this model in other districts across the country with high artisanal and small-scale mining activity.

## Figures and Tables

**Table 1:** ASMs screened for TB, HIV and silicosis at the OHCs and WBS.

	Screened for TB	TB Cases	Bacteriologically Confirmed TB *n* (%) ^†^	Screened for Silicosis	Silicosis Cases *n* (%) ^††^	Eligible for TPT	On TPT *n* (%) ^†††^	Silico-TB Cases	HIV-Positive
October 2020–September 2021	1965	111	43 (39)	1857	330(18)	302	242 (80)	31	201
October 2021–September 2022	5298	350	113 (32)	5147	863 (17)	640	414 (65)	223	449
October 2022–September 2023	3405	332	158 (48)	3278	957 (29)	723	556 (77)	220	341
Total	10,668	793	314 (40)	10,282	2150 (21)	1665	1212 (73)	474	991

^†^ = Percentage of bacteriologically confirmed TB cases among all ASMs diagnosed with TB; ^††^ = percentage of silicosis cases among those who underwent screening for silicosis; ^†††^ = percentage of ASMs started on TPT among those who were eligible for TPT.

**Table 2:** ASMs screened at the OHCs.

	Screened for TB	TB Cases	Bacteriologically Confirmed TB Cases *n* (%) ^†^	Screened for Silicosis	Silicosis Cases *n* (%) ^††^	Eligible for TPT	On TPT *n* (%) ^†††^	Silico-TB Cases	HIV-Positive
October 2020–September 2021	524	85	33(39)	524	88 (17)	74	51 (69)	14	111
October 2021–September 2022	1372	278	87 (31)	1372	406 (30)	218	118 (54)	188	182
October 2022–September 2023	1557	304	145 (48)	1409	562 (39)	349	265 (76)	200	285
Total	3453	667 (19)	265 (40)	3305	1056 (32)	641	434 (68)	402	578

^†^ = Percentage of bacteriologically confirmed TB cases among all ASMs diagnosed with TB; ^††^ = percentage of silicosis cases among those who underwent screening for silicosis; ^†††^ = percentage of ASMs started on TPT among those who were eligible for TPT.

**Table 3:** ASMs screened for TB, HIV and silicosis at WBS services.

	Screened for TB	TB Cases	Bacteriologically Confirmed *n* (%) ^†^	Screened for Silicosis	Silicosis Cases *n* (%) ^††^	Eligible for TPT	On TPT *n* (%) ^†††^	Silico-TB Cases	HIV-Positive
October 2020–September 2021	1441	26	10 (38)	1333	242 (18)	228	191 (84)	17	90
October 2021–September 2022	3926	72	26 (36)	3775	457 (12)	422	296 (70)	35	267
October 2022–September 2023	1848	28	13 (46)	1869	395 (21)	374	291 (78)	20	56
Total	7215	126	49 (39)	6977	1094 (16)	1024	778 (76)	72	413

^†^ = Percentage of bacteriologically confirmed TB cases among all ASMs diagnosed with TB; ^††^ = percentage of silicosis cases among those who underwent screening for silicosis; ^†††^ = percentage of ASMs started on TPT among those who were eligible for TPT.

## Data Availability

The data presented in this study are available on request from the corresponding author. The data are not publicly available due to authorizations that may be required by the Ministry of Health and Childcare of Zimbabwe.
